# New findings useful for clinical practice using swept-source optical coherence tomography angiography in the follow-up of active ocular toxoplasmosis

**DOI:** 10.1186/s40942-020-00231-2

**Published:** 2020-07-08

**Authors:** João Rafael de Oliveira Dias, Camila Campelo, Eduardo Amorim Novais, Gabriel Costa de Andrade, Paula Marinho, Yusláy Fernández Zamora, Luciana Finamor Peixoto, Maurício Maia, Heloísa Nascimento, Rubens Belfort

**Affiliations:** 1 Department of Ophthalmology, Federal University of São Paulo, Paulista Medical School, São Paulo, SP Brazil; 2 Vision Institute, São Paulo, SP Brazil; 3 Rua Marechal Bormann, 243-E., Chapecó, SC 89802-120 Brazil

**Keywords:** Ocular toxoplasmosis, Optical coherence tomography angiography, Toxoplasmic retinochoroiditis

## Abstract

**Background:**

Ocular toxoplasmosis is one of the most common causes of intraocular inflammation and posterior uveitis in immunocompetent patients. This paper aims to investigate swept-source optical coherence tomography angiography (SS-OCTA) findings in eyes with active toxoplasmic retinochoroiditis.

**Methods:**

This case series was conducted from November 2017 through October 2019 in two Brazilian centers. 15 eyes of 15 patients with active toxoplasmic retinochoroiditis were included, and were imaged at baseline and after at least 4 weeks of follow-up. All patients underwent ophthalmic examinations and multimodal imaging including SS-OCT and SS-OCTA before and after treatment of ocular toxoplasmosis. The differential diagnoses included toxoplasmosis, syphilis, and human immunodeficiency virus, which were eliminated through serologic and clinical evaluations.

**Results:**

All 15 patients presented with positive anti-*Toxoplasma gondii* immunoglobulin G titers and three also presented with positive anti-*T. gondii* immunoglobulin M titers. The mean age at examination was 32.4 years ± 12.7 years (range 15–59 years). Sixty percent of the patients were female. In all eyes, the inner retinal layers were abnormally hyperreflective with full-thickness disorganization of the retinal reflective layers at the site of the active toxoplasmic retinochoroiditis. At baseline, 80% of eyes had focal choroidal thickening beneath the retinitis area, and all eyes had a choroidal hyporeflective signal. Before treatment, SS-OCTA showed no OCTA decorrelation signal next to the lesion site in all eyes, and flow signal improvement was noticed after treatment. Three eyes presented with intraretinal vascular abnormalities during follow-up. SS-OCTA showed retinal neovascularization in one patient and a presumed subclinical choroidal neovascular membrane in another patient.

**Conclusions:**

SS-OCT and SS-OCTA are useful for assessing unexpected structural and vascular retinal and choroidal changes in active and post-treatment toxoplasmic retinochoroiditis and these findings are useful for clinical practice.

## Introduction

Toxoplasmosis, caused by the protozoan *Toxoplasma gondii*, is one of the most common causes of intraocular inflammation and posterior uveitis in immunocompetent patients [1, 2]. The infection results from ingestion of undercooked or raw meat containing *T. gondii* cysts and water or food containing oocysts excreted in the feces of infected cats [3, 4]. Infections that occur during pregnancy can result in abortion and fetal death or congenital toxoplasmosis, which includes neurologic and neurocognitive deficits and retinochoroiditis in the newborn [5–7]. Acquired toxoplasmosis often is asymptomatic in immunocompetent individuals, but ocular lesions may be present in up to 20% of infected individuals in certain regions of the world [8]. Focal necrotizing retinitis associated with vitreous and anterior chamber inflammation is the hallmark of ocular toxoplasmosis (OT) [9]. A severe vitritis causes the classic “headlight in the fog” sign [10].

Optical coherence tomography (OCT) is a noninvasive imaging technique that effectively detects pathologic features in uveitis [11, 12]. Commercially available swept-source (SS) OCT uses a 1050-nm wavelength and can penetrate ocular opacities such as cataract and vitritis, which facilitates better retinal and choroidal visualization compared to 840-nm-wavelength spectral-domain (SD) technology [13, 14]. OCT angiography (OCTA) uses motion contrast imaging to obtain high-resolution volumetric blood flow information to generate angiographic images, which provides an accurate definition of the microvasculature of the retinal and choroidal layers and identification of abnormalities [15]. En face OCTA images can be scrolled outward from the internal limiting membrane up to the choroid to assess the individual vascular plexus and segment the inner and outer retina, choriocapillaris, and choroid [16]. The current study investigated the structural and angiographic SS-OCT findings in eyes with active OT.

## Methods

Patients with active OT were enrolled from two centers, i.e., São Paulo and Chapecó cities in southeastern and southern Brazil from November 2017 through October 2019 in this prospective, consecutive OCT-imaging study. The institutional review boards of the Federal University of São Paulo, Paulista Medical School (IRB number, 3.445.576), and the Vision Institute (IRB number, 3.476.558) approved the study; all patients provided informed consent before entry into the study. The study was performed in accordance with the tenets of the Declaration of Helsinki and complied with the Health Insurance Portability and Accountability Act of 1996.

The inclusion criteria were a diagnosis of active OT that was based on the presence of one or more focal white retinal lesions with or without adjacent hyperpigmented retinal and choroidal scars and confirmed by anti-Toxoplasma antibody analysis. All patients were tested for anti-*T. gondii* immunoglobulin G (IgG) and immunoglobulin M (IgM). The exclusion criteria were patients with positive serum analysis for syphilis and human immunodeficiency virus tests and inadequate imaging or the absence of follow-up. The same examiners (RBJ in São Paulo and JROD in Chapecó) performed fundus and SS-OCT with SS-OCTA imaging for each patient during the same imaging session at baseline and during various periods of follow-up (4–18 weeks).

The SS-OCT devices (DRI OCT Triton, Topcon, Tokyo, Japan), located in São Paulo and Chapecó cities, were used to obtain the OCT images. The OCT uses a 1050-nm wavelength with an acquisition speed of 100,000 A-scans/s. The scans were performed in 3 × 3-, 6 × 6-, and 9 × 9-mm fields of view centered on the active toxoplasmic lesion and the macula. The automated layer segmentation mode of the OCT instrument software was used to generate en face images of the retinal vasculature from the superficial and deep retinal layers through en face slabs. Manual correction of automatic segmentation was applied when necessary. The superficial retinal layer was defined as extending from the internal limiting membrane to the inner plexiform layer, and the deep retinal layer was defined as extending from the inner plexiform layer to the outer plexiform layer. The avascular retina was defined as extending from the layer between the outer plexiform layer in the inner border and inner segment/outer segment junction in the outer border. The OCTA images were processed using the amplitude referred to as the OCTA ratio analysis, which is a motion contrast measure using a ratio method.

Two scans were performed in each eye, and the scan with the best signal strength was used. Scans were excluded from the analyses if the image quality was poor, artifacts caused significant signal blockage such as vitreous opacities; residual motion artifacts were visible as irregular vessel patterns or disc boundaries on the en face angiograms, or peripheral active toxoplasmic lesions could not be evaluated properly. All scans were performed at baseline and up to 18 weeks after treatment. The image quality scores given by the SS-OCT equipment were recorded.

Data analyses were performed using SPSS for Windows, version 22.0 (IBM Corp., Armonk, NY). Continuous variables were analyzed and expressed as the mean and standard deviation.

## Results

Active OT was identified in 24 consecutive patients based on clinical examinations and laboratory results. One patient declined study participation and, in five patients, media opacities and/or the lesion location (zone 3) prevented acquisition of adequate SS-OCT images. Three patients were excluded due to the absence of follow-up. Ultimately, 15 patients were included, three of whom presented with positive anti-toxoplasmosis IgM titers. The mean age at examination was 32.4 ± 12.7 years (range 15–59 years). Sixty percent of the patients were female.

All patients had mild to moderate vitritis at baseline. Vitritis was graded by the examiners in 1+ to 4+. One of the patients (*patient 9*) presented with a 3+ vitritis at baseline and a signal strength of 27 in both 6 × 6-mm and 9 × 9-mm SS-OCTA fields of view. In spite of the lower signal strength the analysis and interpretation of the images was possible. All patients except patient 5 presented with anterior uveitis at baseline, however, it did not prevent the acquisition of OCTA images. None of the patients presented with cataract at baseline or follow-up. Table 1 summarizes the patients’ characteristics, serologic status, and ophthalmologic examinations at baseline and during follow-up.

**Table 1:** Characteristics of patients with active ocular toxoplasmosis

Patient no., age, gender, study eye	Symptom onset^a^	Lesion location (zone)	Lesion size (DD)	BCVA at baseline	BCVA at follow-up	IgM	IgG	Clinical findings
1, 29 years, F, OD	20 days	1	2–3	Hand motion	20/125	NR	R	ACC 3+, IRH, VC 2+, IRL
2, 25 years, M, OS	15 days	2	1	20/20	20/20	NR	R	ACC1+, RNV, VC1+
3, 22 years, M, OS	1 day	1	< 1	20/60-2	20/25-1	NR	R	ACC 3+, KP, VC 1+, IRL, CNVM
4, 29 years, F, OD	15 days	1	< 1	20/160	20/120-1	R	R	ACC 3+, VC3+, IRL
5, 20 years, M, OD	4 days	1	< 1	20/25-2	20/20	NR	R	VC 2+
6, 31 years, F, OS	20 days	1	< 1	20/32	20/20	NR	R	ACC 1+, VC 2+
7, 20 years, F, OD	5 days	1	1–2	20/125	20/40	NR	R	ACC 3+, IRH, VC 1+
8, 15 years, F, OD	10 days	1	1–2	20/32-2	20/20	NR	R	ACC 1+, VC 1+
9, 40 years, M, OD	21 days	1	2–3	20/500	20/334	NR	R	ACC 3+, KP, VC 3+
10, 56 years, F, OS	6 days	1	< 1	Hand motion	20/30	NR	R	ACC 3+, KP, VC 2+
11, 41 years, F, OD	15 days	1	1–2	20/160	20/125	R	R	ACC 2+, VC 2+
12, 39 years, F, OD	5 days	1	1–2	20/100	20/32	NR	R	ACC 3+, VC1+
13, 28 years, M, OS	6 days	1	3	20/334	20/40	NR	R	ACC 3+, KP, VC 1+
14, 59 years, F, OS	15 days	1	> 6 DP	20/125	20/40	NR	R	ACC 3+, VC 2+
15, 32 years, M, OD	5 days	1	1–2	CF 1 m	20/100	R	R	ACC 2+, VC 1+

*DD* disc diameter, *BCVA* best-corrected visual acuity, *CF* counting fingers, *IgG* toxoplasmosis immunoglobulin G, *IgM* toxoplasmosis immunoglobulin M, *F* female, *M* male, *OD* right eye, *OS* left eye, *R* reagent, *NR* non-reagent, *ACC* anterior chamber cells, *VC* vitreous cells, *IRH* intraretinal hemorrhage, *RNV* retinal neovascularization, *KP* keratic precipitates, *CNVM* choroidal neovascular membrane, *IRL* intraretinal loops

^a^Time interval between symptom onset and baseline evaluation

All patients had negative serology results for syphilis and human immunodeficiency virus. Fourteen patients were treated with 800 mg of oral sulfamethoxazole and 160 mg of trimethoprim (Bactrim^®^F, Roche Pharmaceuticals, Rio de Janeiro, Brazil) twice daily (bid) for 6 weeks or according to ophthalmologic criteria, and 0.5 mg/kg/day of prednisone tapered over 4 to 6 weeks [17]. One patient who was allergic to sulfamethoxazole was treated with 300 mg of clindamycin three times daily, pyrimethamine (loading dose, 50 mg for 4 days; treatment dose, 25 mg daily), 5 mg daily of folinic acid, and 0.5 mg/kg/day of prednisone as already reported [18] (patient 4 in Table 1).

### Structural SS-OCT B-scan findings

**Fig. 1:**
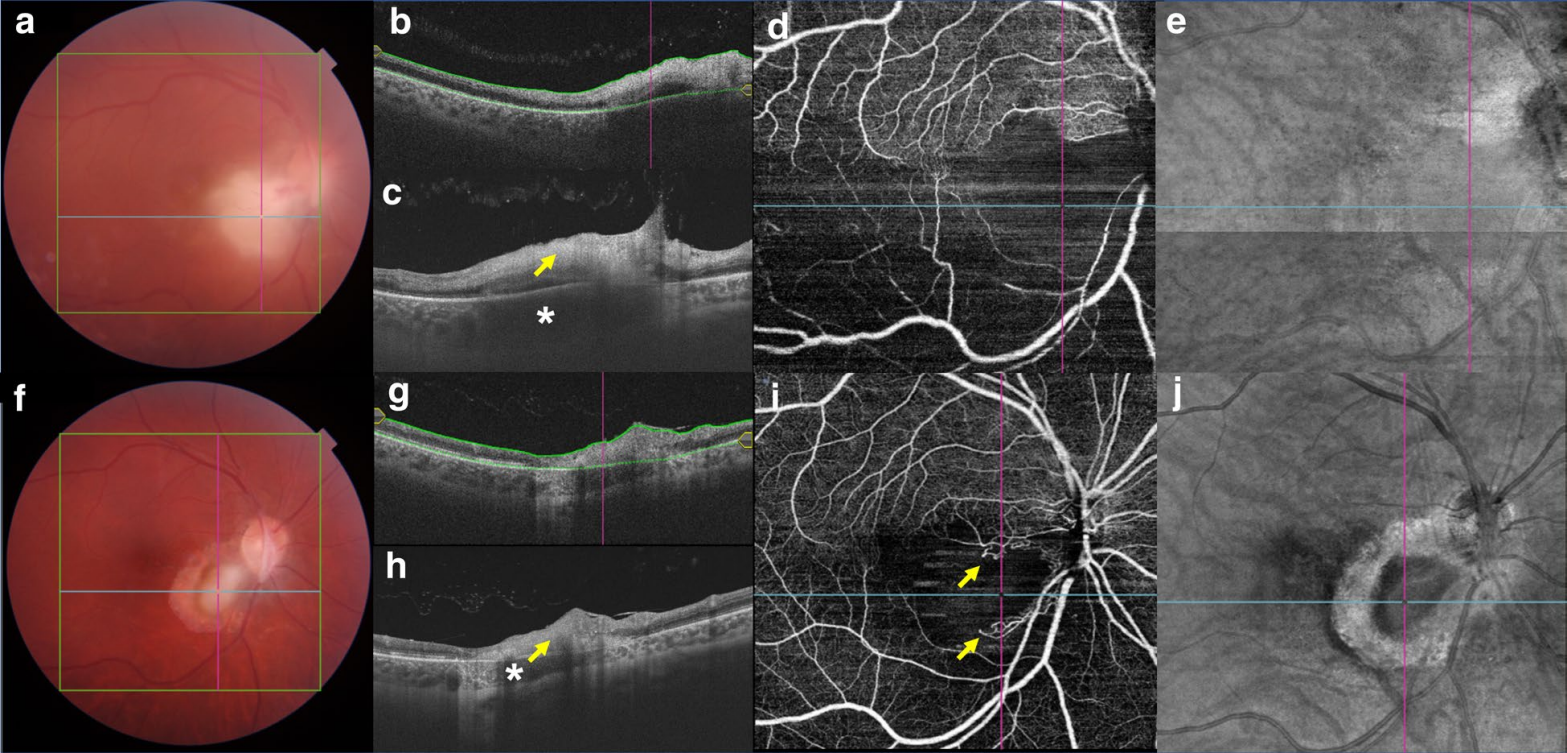
**a** Color fundus at the time of diagnosis. Vitritis and an active toxoplasmic lesion are visible in the papillomacular bundle. **b** Optical coherence tomography (OCT) B-scan with the total retina slabs. **c** OCT B-scan over the active lesion shows retinal hyperreflectivity (arrow) and a thickened choroid (asterisk) under the lesion. **d** A 9 × 9-mm field of view swept-source OCTA (SS-OCTA) image shows inferiorly located no OCTA decorrelation signal, suggestive of reduced blood flow. **e** Structural en face image. **f** Color fundus image 13 weeks after those in (**a**–**e**): inflammatory signs are absent, but an atrophic scar is visible. **g** OCT B-scan with the total retina slabs. **h** Retinal layers are unrecognizable at the lesion site (arrow) and choroidal thinning is seen (asterisk). **i** A 9 × 9-mm field of view on SS-OCTA shows increase in flow signal after treatment. The arrows indicate the vascular loops. **j** A structural en face image

**Fig. 2:**
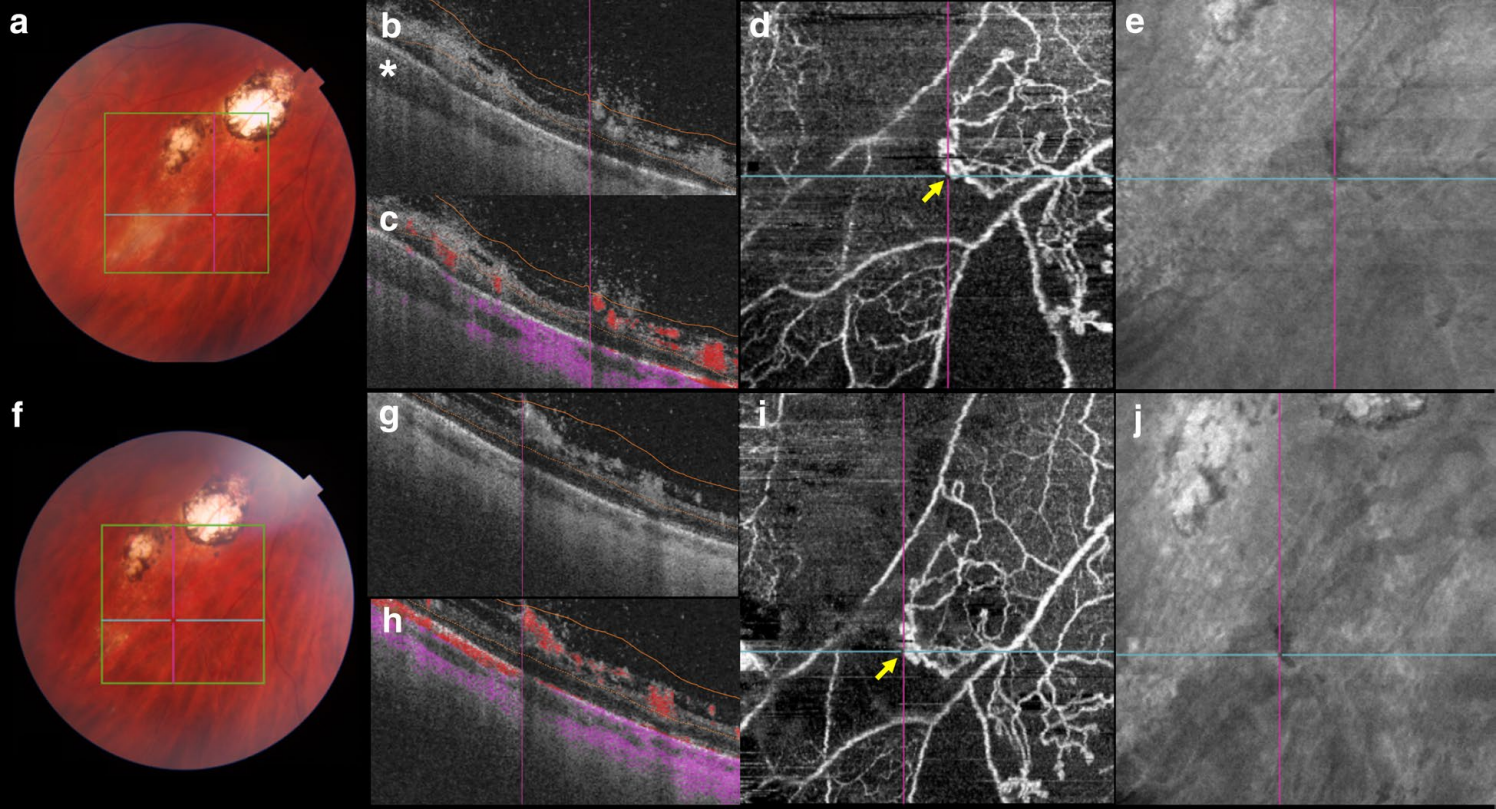
**a** Color fundus at the time of diagnosis. An active toxoplasmic lesion is visible adjacent to the retinal choroidal pigmented scars. **b** An optical coherence tomography (OCT) B-scan with slabs extending from the vitreous to the inner nuclear layer. Thickened choroidal tissue is seen adjacent to the lesion site (asterisk). **c** An OCT B-scan with overlying color-coded flow in which red represents the retinal capillaries and pre-retinal neovascularization, and pink represents the choroid. **d** A 6 × 6-mm field of view on OCT angiography (OCTA) shows a neovascular seafan-like complex (arrow) and areas of no decorrelation signal. **e** A structural en face OCT shows a hyporeflective lesion suggestive of retinal neovascularization. **f** A color fundus image obtained 5 weeks after those in (**a**–**e**) shows that the area of retinitis has regressed following ocular toxoplasmosis treatment. **g** An OCT B-scan with slabs extending from the vitreous to the inner nuclear layer. **h** An OCT B-scan with overlying color-coded flow shows that the retinal neovascularization appears unchanged after the systemic anti-Toxoplasma treatment. **i** A 6 × 6-mm OCTA image shows unchanged retinal neovascularization (arrow). **j** A structural en face OCT image

**Fig. 3:**
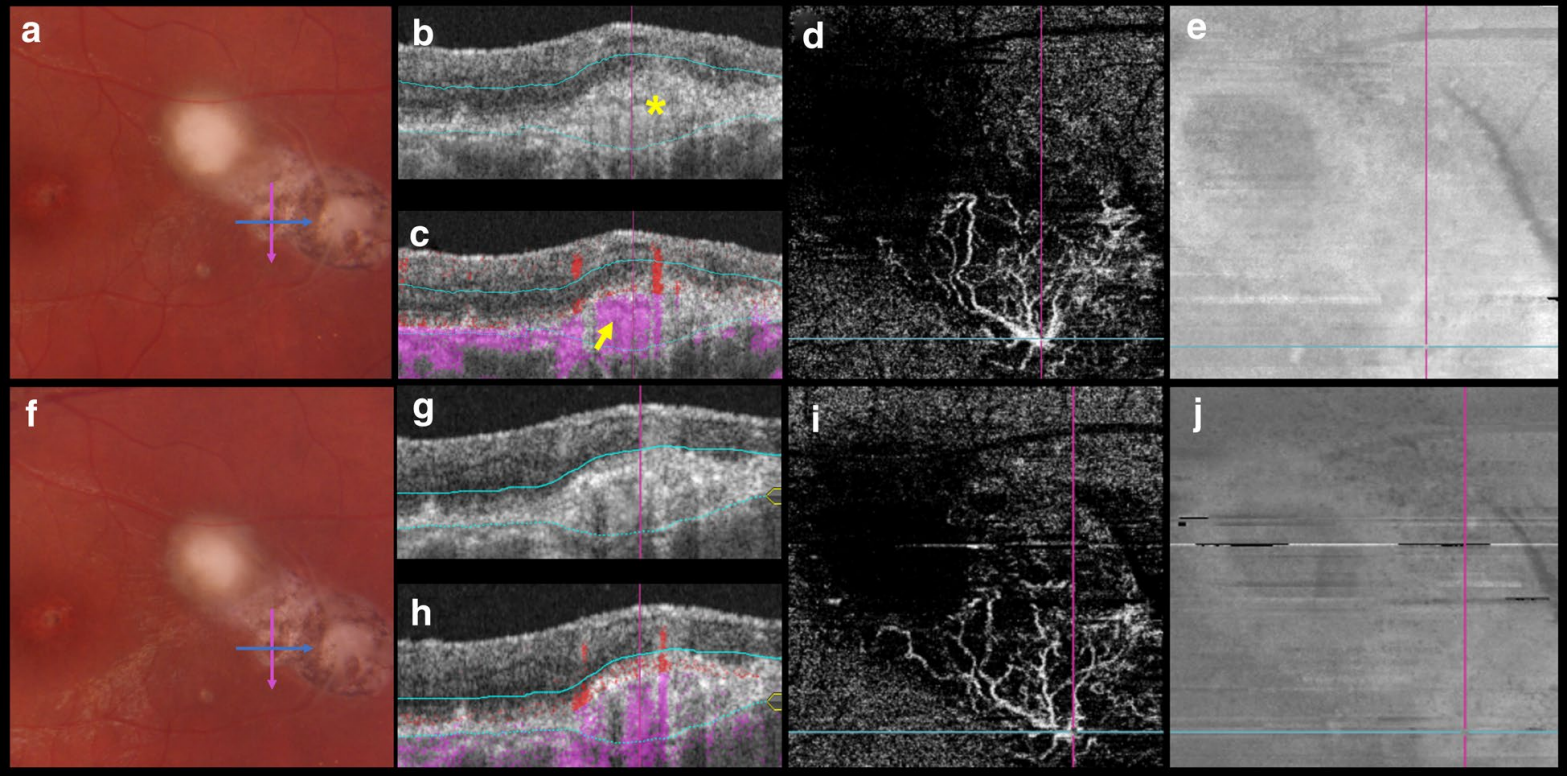
**a** Color fundus 6 weeks after treatment. The pink and blue arrows indicate the B-scan positions in B and C. **b** An optical coherence tomography (OCT) B-scan with the outer retina slabs. Hyperreflective material is seen in the subretinal space (asterisk). **c** An OCT B-scan with overlying color-coded flow in which red represents the retinal capillaries, and pink represents the choroid and the choroidal neovascular membrane (CNVM). **d** A 3 × 3-mm field of view on a swept-source OCT angiography (SS-OCTA) image suggests the presence of a CNVM. **e** A structural en face OCT shows hyporeflective dots in the CNVM location. **f** Color fundus image 4 weeks after those in (**a**–**e**). **g** An OCT B-scan with the outer retina slabs. **h** An OCT B-scan with overlying color-coded flow. **i** A 3 × 3-mm OCTA image showing the CNVM. **j** A structural en face image

All patients had classic presumed toxoplasmic focal fundus lesion(s) (Figs. 1a, 2a, 3a) with an adjacent retinal choroidal scar in 33% of the eyes (Figs. 2a, 3a). At baseline, all eyes had punctate hyperreflective spots in the posterior vitreous representing a collection of inflammatory material and inflammatory cells. Fewer vitreous cells were observed after treatment. Preretinal oval deposits overlying areas of retinitis were observed in four (27%) eyes.

At baseline, all patients had increased retinal reflectivity and thickness associated with disorganization of the neuroretinal layer boundaries at the active lesion site (Fig. 1b, c). Perilesional subretinal fluid was seen in 20% of the eyes with active OT. Twelve (80%) eyes had focal choroidal thickening beneath the area of retinitis, and all eyes had a choroidal hyporeflective signal (Figs. 1c, 2b). A retinal choroidal scar with a disrupted, thinning choroid beneath a thin hyperreflective retina was observed after treatment, and the disorganized retinal layer reflectivity due to scar formation remained in all eyes (Fig. 1g, h).

### SS-OCTA findings

For the 3 × 3-mm fields of view, the image quality scores varied from 47 to 64 at baseline and from 56 to 65 at follow-up. For the 6 × 6-mm fields of view the image quality scores varied from 27 to 69 at baseline and from 35 to 68 at follow-up. For the 9 × 9-mm fields of view the image quality scores varied from 27 to 68 at baseline and from 40 to 66 at follow-up. The lower signal at baseline was probably because of media opacity secondary to active uveitis.

The 3 × 3-, 6 × 6-, and 9 × 9-mm OCTA fields of view obtained during the active disease stage at the lesion site showed no OCTA decorrelation signal, suggestive of reduced blood flow (Fig. 1d). After healing OCTA images showed persistence of reduced flow signal in eleven patients with partial regression of the previously observed hyporeflective signal and improved visualization of the normal architecture of the vascular retinal layers (Fig. 1i). The superficial and deep retinal capillary plexuses and the choriocapillaris could have been affected by the disorganization of the neurosensory retina due to toxoplasmosis retinochoroiditis.

One patient presented with retinal neovascularization at baseline (Fig. 2a–e) which remained unchanged after treatment (Fig. 2f–j). Another patient was diagnosed after 6 weeks of OT treatment with a presumed subclinical choroidal neovascular membrane (CNVM) (Fig. 3d). Three patients presented with intraretinal vascular abnormalities at baseline during follow-up that were seen on the OCTA images (1i). Table 2 summarizes the SS-OCTA main results.

**Table 2:** SS-OCT and SS-OCTA characteristics of patients with active ocular toxoplasmosis

Patient no.	Thickened choroid below retinochoroiditis		Hyporeflective choroidal signal below retinochoroiditis		Reduced decorrelation signal adjacent to retinochoroiditis		Intraretinal abnormalities		Retinal neovascularization		Choroidal neovascular membrane	
	Baseline	FU	Baseline	FU	Baseline	FU	Baseline	FU	Baseline	FU	Baseline	FU
1	X		X	X	X	X		X				
2	X		X	X	X	X			X	X		
3	X		X	X	N/A	X	N/A	X	N/A		N/A	X
4	X		X		X							
5			X		X	X						
6	X		X		X	X		X				
7	X		X	X	X	X						
8	X		X		X							
9	X		X	X	X	X						
10			X	X	X	X						
11			X	X	X	X						
12	X		X	X	X							
13	X		X	X	X	X						
14	X		X		X	X						
15	X		X	X	X							

*FU* follow-up, *N/A* not available

### Report of cases

#### Patient 1

A 29-year-old healthy woman complained of visual loss in her right eye (hand motion vision) for 20 days (patient 1 in Tables 1 and 2). The visual acuity (VA) in the left eye was 20/20. The results of anterior biomicroscopy of the right eye showed keratic precipitates, 3+ anterior chamber cells, and 2+ anterior vitreous cells. A fundus examination showed 2+ vitritis and a focal necrotizing retinal choroidal lesion with a retinal hemorrhage in the papillomacular bundle (Fig. 1a). A SS-OCT image obtained through the active lesion showed nerve fiber swelling and choroidal thickening (Fig. 1b, c). The OCTA performed on the lesion showed no decorrelation signal (Fig. 1d), suggestive of reduced blood flow. The clinical suspicion of toxoplasmosis was confirmed by positive IgG levels (35.10 IU/ml; reference value, < 1.6 IU/ml). The IgM value was borderline (0.45 IU/ml; reference value, < 0.5 IU/ml). The patient was treated with an oral combination of 160 mg of trimethoprim and 800 mg of sulfamethoxazole, 1 tablet bid for 6 weeks, and 40 mg/day of prednisone tapered over 6 weeks which resulted in lesion regression (Fig. 1f) and VA improvement (20/125). After healing, SS-OCT imaging of the lesion showed disorganization of the entire retinal architecture. The borders of the retinal layers in the retinal choroidal lesion were unrecognizable (Fig. 1g, h). The choroid surrounding the lesion site was thinner compared to the adjacent areas (Fig. 1h). An OCTA image of the lesion showed increase in flow signal and anomalous vessels in the retinochoroiditis area (Fig. 1i).

#### Patient 2

A 25-year-old healthy man presented with blurred vision in his left eye (VA, 20/20) for 15 days (patient 2 in Tables 1 and 2). The right eye had 20/40 VA due to amblyopia. He reported previous treatments for OT. Anterior biomicroscopy of the left eye showed 1+ anterior chamber cells. A fundus examination showed mild vitritis, a focal exudative retinal choroidal lesion inferior to the pigmented retinal choroidal scars (Fig. 2a). A SS-OCT image through the active lesion showed that the inner retinal layers were abnormally hyperreflective with full-thickness disorganization of the retinal reflective layers. The choroid appeared thickened next to the active lesion (Fig. 2b). The color-coded decorrelation signal that overlaid the OCTA B-scan performed next to the lesion site showed no OCTA decorrelation signal and retinal neovascularization (Fig. 2c–e), which were confirmed by fluorescein angiography (FA). The clinical suspicion of toxoplasmosis was confirmed by high positive IgG levels (461.7 IU/ml; reference value, < 30 IU/ml). The IgM titer was negative (< 0.8 IU/ml). The patient was treated with an oral combination of 160 mg of trimethoprim and 800 mg of sulfamethoxazole, 1 tablet bid for 6 weeks, which resulted in lesion regression (Fig. 2f) and resolution of the visual symptoms. OCTA performed next to the lesion site showed persistent absence of decorrelation signal around the unchanged retinal neovascularization (Fig. 2h–j). The patient underwent sectorial retinal photocoagulation during this same visit.

#### Patient 3

A 22-year-old healthy man reported blurred vision in his left eye (VA, 20/60^−2^) for 1 day (patient 3 in Tables 1 and 2). The right eye had 20/250 VA due to a macular retinal choroidal scar. The results of anterior biomicroscopy of the left eye showed fine keratic precipitates and 3+ anterior chamber cells. A fundus examination showed mild vitritis and a parafoveolar focal necrotizing retinal choroidal lesion next to the retinal choroidal scars. Structural SS-OCT images were obtained, but poor fixation prevented the acquisition of SS-OCTA images at baseline. The clinical suspicion of toxoplasmosis was confirmed by positive IgG levels (19.7 IU/ml; reference value, < 1.6 IU/ml). The IgM titer was negative (0.06 IU/ml). The patient was treated with an oral combination of 160 mg of trimethoprim and 800 mg of sulfamethoxazole, 1 tablet bid for 6 weeks, and 60 mg daily of prednisone that was tapered over the ensuing weeks, which resulted in lesion regression (Fig. 3a) and resolution of the visual symptoms. SS-OCT performed 6 weeks after treatment through the retinal choroidal scar showed a subretinal area of hyperreflectivity extending toward the choroid (Fig. 3b, c), which was highly suggestive of a subclinical CNVM on SS-OCTA (Fig. 3d). FA imaging showed areas of hyperfluorescence without leakage at the borders of the retinal choroidal lesion. Indocyanine green angiography was not performed because it was unavailable at the study site. An OCTA image of the lesion site showed abnormal intraretinal loops surrounding the retinochoroiditis lesion and partial flow signal of the retinal. SS-OCT and SS-OCTA images were obtained 4 weeks after the treatment end (Fig. 2f–j).

## Discussion

SS-OCT combined with en face and OCTA technologies is a non-invasive imaging technology that is particularly useful for assessing patients with active OT, because it allows visualization of the vitreous and all retinal layers up to the choroid. Despite the presence of vitritis, this approach also provides better images compared with SD and time-domain OCTs [14]. SS-OCT also offers high imaging speeds, high detection efficiencies, improved imaging range, and improved depth with reduced sensitivity roll-off. Those features contribute to the improved image quality, including that of the choroid [19, 20]. In this present case series SD-OCT and SD-OCTA were not performed as these imaging modalities were available in only one of the study sites. Although a comparison between these two OCT modalities would be interesting, we believe that SS-OCT and SS-OCTA provide better information in comparison do SD-OCT and SD-OCTA, as previous reports have shown that SS-OCT has less sensitivity roll-out and better visualization through media opacities when compared to SD-OCT [21].

The posterior segment findings during acute and healed toxoplasmic retinochoroiditis have been well described using SD-OCT [22–26]. During the acute phase, multiple hyperreflective dots are seen in the vitreous cavity. Other findings include posterior hyaloid thickening and subsequent separation and migration of inflammatory cells into the vitreous and spherical deposits that appear along the retinal vessels [24, 27]. During the active disease stages, the choroid is also characterized by focal thickening and loss of the physiologic architecture and becomes homogenously hyporeflective. The technology allows differentiation of the toxoplasmic foci of necrotizing retinitis from those with a viral etiology that are characterized by a normal-appearing choroid [27]. In the current study, choroidal thickening during the active phase was seen in 80% of patients, and evolution to choroidal thinning was seen in all patients after resolution of the disease. The exact cause of choroidal thickening cannot be ascertained without histologic confirmation. However, a reasonable speculation is that choroidal thickening may result from inflammatory cell infiltration and increased blood flow [28]. Case reports have analyzed SS-OCT findings in active retinochoroiditis [14] including with SS-OCTA technology [29–33]. Vezzola et al. [29] published for the first time OCTA images of active OT from the acute to the quiescent stages. Those authors believe that after healing the vascular obliteration appears to be only partially reversible in the periphery of the lesion and not in the center. In addition, the choriocapillaris destruction persists around the obliterated area and only the deep choroidal vessels remain visible [29]. All of the current eyes, with exception of patient 3 who did not perform SS-OCTA images at baseline, had signal blockage in the acute phase. However, it remains unclear if the signal decrease during the acute phase is due to vascular obliteration or masking of the superficial capillary plexus, deep capillary plexus, and choriocapillaris by the focal lesion.

Intraretinal abnormalities are another feature that have been described recently in patients with toxoplasmic retinochoroiditis [32]. Using OCTA, Toutée et al. first reported two cases of intraretinal abnormal vascular processes associated with acute Toxoplasma retinitis. The authors suggested that vascular changes may correspond to vascular remodeling within a necrotic retina due to an inflammatory lesion [32]. Three (20%) of the current patients presented with vascular loops after treatment, at the retina, indicating the presence of an intraretinal vascular process.

Retinal neovascularization also has been described previously in OT. Rodríguez et al. reported long-lasting obstructive effects on the return venous flow crossing plaques of cicatricial toxoplasmic retinochoroiditis that have been observed in association with distal sprouts of retinal neovascularization in the fundus [34]. One current patient presented with retinal neovascularization seen on OCTA images, which was confirmed in FA images. This patient underwent sectorial laser photocoagulation to prevent vitreous hemorrhage and tractional retinal detachment. OCTA definitely played an important role in the diagnosis of retinal neovascularization, because on this patient specific case the retinal new vessels were not promptly recognized during routine indirect ophthalmoscopy. Besides, fluorescein angiography is not routinely performed in patients with ocular toxoplasmosis because of the risk of adverse events, inconvenience, and discomfort associated. To our knowledge, the current study is the first to document retinal neovascularization using OCTA in an eye with active toxoplasmic retinochoroiditis.

CNVM has been reported at the margins of the healed Toxoplasma scars in 2% to 19% of cases [35, 36]. Although this entity is uncommon during active retinochoroiditis, it has been described previously [37, 38]. Case reports about CNVM in toxoplasmosis have confirmed the use of OCTA for CNVM secondary to OT, especially because FA findings can be difficult to interpret in a scarred area [26, 39, 40]. Previous OCTA studies have described subclinical type 1 macular neovascularization (MNV) in patients with age-related macular degeneration (AMD), chronic central serous chorioretinopathy and polypoidal choroidal vasculopathy [41–43]. Although structural B-scan can suggest the presence of the MNV, direct visualization of these non-exudative neovascular lesions requires the use of ICGA or OCTA [44]. A majority of inflammatory CNVMs are type 2 lesions with abnormal growth of vasculature into the outer retinal space [45]. However, inflammatory CNVM does not necessarily present with a sub- or intraretinal fluid on OCT and may just show an increase in subretinal hyperreflectivity [40]. One current patient presented with a subclinical type 2 CNVM adjacent to a toxoplasmic lesion. Because the CNVM did not show signs of exudation, a conservative approach was adopted, and anti-vascular endothelial growth factor injections were not indicated. The patient will be followed closely because of the risk of CNVM exudation. Although subclinical CNVM has already been described with SS-OCTA in eyes with multifocal choroiditis [40, 46], to our knowledge the current study is the first to describe a subclinical CNVM in a patient with OT.

The current study is the largest investigation to date of patients with active OT that underwent SS-OCTA at baseline and during follow-up. Three patients had a primary systemic toxoplasmosis infection and showed positive anti-Toxoplasma IgM titers. We also reported for the first time the unique features of OT seen in the OCTA images, including retinal neovascularization and a lesion that was presumed to be a subclinical CNVM in an eye with active OT.

This study had some limitations, including that only one type of SS-OCT device was used. However, a comparison between different SS-OCTs was outside of the scope of this study. In addition, the patients were imaged during follow-up periods of different lengths because the study was conducted in different centers, which could have affected the results. Besides, in one of the patients (patient 3) SS-OCTA was only recorded after 6 weeks of treatment because poor fixation prevented the acquisition of OCTA images at baseline.

## Conclusions

SS-OCT combined with en face and angiography technologies is an excellent tool for assessing the retinal and choroidal structural and vascular changes and can be used to monitor treatment responses in active OT.

## Data Availability

The datasets used and/or analyzed during the current study are available from the corresponding author on reasonable request.

## Abbreviations

OT: Ocular toxoplasmosis
OCT: Optical coherence tomography
SS: Swept-source
SD: Spectral-domain
OCTA: OCT angiography
IRB: Institutional review boards
IgG: Immunoglobulin G
IgM: Immunoglobulin M
CNVM: Choroidal neovascular membrane
VA: Visual acuity
FA: Fluorescein angiography
DD: Disc diameter
BCVA: Best-corrected visual acuity
CF: Counting fingers
F: Female
M: Male
OD: Right eye
OS: Left eye
R: Reagent
NR: Non-reagent
ACC: Anterior chamber cells
VC: Vitreous cells
IRH: Intraretinal hemorrhage
RNV: Retinal neovascularization
KP: Keratic precipitates
IRL: Intraretinal loops
